# Informed decision-making with and for people with dementia – efficacy of the PRODECIDE education program for legal representatives: protocol of a randomized controlled trial (PRODECIDE-RCT)

**DOI:** 10.1186/s12877-017-0616-z

**Published:** 2017-09-15

**Authors:** Julia Lühnen, Burkhard Haastert, Ingrid Mühlhauser, Tanja Richter

**Affiliations:** 1 Unit of Health Sciences and Education, Faculty of Mathematics, Informatics and Natural Sciences (MIN), University of Hamburg, Martin-Luther-King-Platz 6, 20146 Hamburg, Germany; 2 mediStatistica, Lambertusweg 1b, 58809 Neuenrade, Germany

**Keywords:** Proxy decision-making, Dementia, Legal representatives, Education program, Informed decision, Evidence-based medicine

## Abstract

**Background:**

In Germany, the guardianship system provides adults who are no longer able to handle their own affairs a court-appointed legal representative, for support without restriction of legal capacity. Although these representatives only rarely are qualified in healthcare, they nevertheless play decisive roles in the decision-making processes for people with dementia. Previously, we developed an education program (PRODECIDE) to address this shortcoming and tested it for feasibility. Typical, autonomy-restricting decisions in the care of people with dementia—namely, using percutaneous endoscopic gastrostomy (PEG) or physical restrains (PR), or the prescription of antipsychotic drugs (AP)—were the subject areas trained. The training course aims to enhance the competency of legal representatives in informed decision-making. In this study, we will evaluate the efficacy of the PRODECIDE education program.

**Methods:**

A randomized controlled trial with a six-month follow-up will be conducted to compare the PRODECIDE education program with standard care, enrolling legal representatives (*N* = 216). The education program lasts 10 h and comprises four modules: A, decision-making processes and methods; and B, C and D, evidence-based knowledge about PEG, PR and AP, respectively.

The primary outcome measure is *knowledge*, which is operationalized as the understanding of decision-making processes in healthcare affairs and in setting realistic expectations about benefits and harms of PEG, PR and AP in people with dementia. Secondary outcomes are sufficient and sustainable knowledge and percentage of persons concerned affected by PEG, FEM or AP. A qualitative process evaluation will be performed. Additionally, to support implementation, a concept for translating the educational contents into e-learning modules will be developed.

**Discussion:**

The study results will show whether the efficacy of the education program could justify its implementation into the regular training curricula for legal representatives. Additionally, it will determine whether an e-learning course provides a valuable backup or even alternative learning strategy.

**Trial registration:**

TRN: ISRCTN17960111, Date: 01/06/2017.

## Background

Guardianship is a legal process that transfers decision-making authority over an individual deemed incapable of managing his or her personal and/or financial affairs to another person. Guardianship systems vary widely worldwide by jurisdiction and implementation. In contrast to systems that deny or restrict the legal capacity of a person, the so-called support system allows the person’s legal capacity to remain intact while offering the person support from trusted individuals to make choices [1].

The German guardianship system provides a supportive instrument for the legal protection of adults, without incapacitation or restriction of legal capacity [2]. If an adult is no longer able to handle his or her own affairs due to mental illness or physical and/or mental disability, a legal representative is appointed by court for certain groups of tasks, such as healthcare affairs, that are tailored to the needs of the adult [2, 3]. Approximately 1.3 million people in Germany have been appointed a legal representative [4], of whom 60% are volunteers (family members and others) and 40%, professional representatives [5]. In 65% of the cases, legal representatives are appointed for healthcare affairs [6], with about 20% of professionals due to dementia [5]. Although the legal representatives only rarely have a qualification in the field of healthcare [5], they are nevertheless required to take a major role in decision-making processes for people with dementia [3]. Until now, however, there are no specific authorization criteria and no standardized training courses for legal representatives in Germany [5]. Therefore, core competencies to support healthcare decision-making cannot be presupposed.

Behavioral and psychological symptoms, such as agitation, sleep and appetite changes are common in dementia [7, 8] and may lead to distress in both patients and carers [9]. Interventions such as artificial nutrition via a percutaneous endoscopic gastrostomy (PEG), the use of physical restraints (PR) and antipsychotic drugs (AP) are frequently applied because of anticipated benefits [10–17]. In Germany, the estimated prevalence rates for such interventions on nursing home residents remain high: 5% for PEG [18], 12.5% for PR [18] and almost 30% for AP [17]. There are strong indicators that these autonomy-restricting interventions are more directed towards facilitating nursing care rather than enhancing the quality of life of people with dementia [19]. Evidence for the claimed benefits is weak or controversial, and all interventions have a substantial potential for harm [11, 12, 15, 20–28]. Due to clinical guidelines, interventions are only indicated in exceptional situations [29, 30]. Therefore, it is likely that their use is often not being justified.

The decision-making processes in dementia care are complex and involve different parties, such as physicians, nursing staff, relatives and legal representatives. The attitudes and beliefs of these parties are important factors in explaining these high intervention rates [13, 14]. In order to reduce autonomy-restricting interventions, all parties involved in the decision-making processes should thus be addressed by educational approaches. Several evaluation and implementation studies of educational approaches to reduce PR and AP have been conducted [20, 31–33]. However, legal representatives have not yet been addressed at all.

We developed an education program for legal representatives and tested its feasibility in a pilot study at the University Hamburg, which ran from April 2013 until September 2015 [34]. The aim of the program was to enhance competencies in informed decision processes, as exemplified by the three decisions in the care of people with dementia (PEG, PR and AP). The program was based on the theory of planned behavior and was developed according to the UK Medical Research Council (MRC) evaluation framework as well as methods of evidence-based medicine [35–37]*.* We generated a curriculum based on systematic literature searches and on interviews with voluntary and professional legal representatives and with senior citizens.

In a previous pilot study, we tested the education program for comprehensibility, feasibility, usability and acceptance [34]. We initially conducted eight trainings with 47 legal guardians. The education program was well accepted, and the comprehensibility of contents and materials was rated as high. Participants stated that they developed essential competencies needed to discuss the necessity of PEG, PR and AP in people with dementia, and to aim for alternative interventions. The program appears ready for implementation, but in line with the UK MRC evaluation framework [36], efficacy has to be proven first. To prepare for evaluation in a randomized controlled trial (RCT), we next defined relevant outcome measures and developed appropriate assessment tools. Finally, to pretest the assessment instruments, we carried out the education program five times for a total of 34 legal representatives. Whenever necessary, questionnaires were revised and assessment strategies were optimized.

### Objectives

The main objective of the planned randomized controlled trial is to evaluate the efficacy of the PRODECIDE education program for legal representatives. The key hypothesis is that legal representatives allocated to the education group would achieve a better understanding of decision-making processes and higher levels of realistic expectations regarding probabilities of benefits and harms of PEG, PR and AP to people with dementia compared to the control group.

Understanding the decision-making processes and setting realistic expectations are prerequisites for informed decision-making. Informed and evidence-based decisions may enhance the quality of care of people with dementia and reduce both the overuse and the misuse of autonomy-restricting interventions. Therefore, a further objective is to determine whether the education of legal representatives can result in a clinically relevant reduction of PEG, PR, and AP in persons with dementia.

We expect that implementing the PRODECIDE education program into the regular training offers for legal representatives will be perceived as feasible. To understand barriers and facilitators, a qualitative process evaluation will be performed.

The reporting of this study follows current statements [38, 39].

In parallel to this trial, we will develop a concept for translating the educational contents into e-learning modules, to further support implementation. Web-based learning creates an additional offer that has neither time nor location restrictions. We expect that this alternative could reach people who do not take part in the face-to-face courses. The e-learning modules will be tested for usability. We assume transferability of acceptance and comprehensibility of educational contents.

## Methods/design

### Design

The PRODECIDE-RCT is a randomized controlled superiority trial with two parallel groups, a 1:1 randomization and a six-month follow-up. Together with the trial, qualitative methods will be used to achieve in-depth understanding of the implementation processes. Additional e-learning modules will be developed and tested for usability.

### Setting

The study will mainly take place in northern and eastern Germany in the areas of Hamburg, Schleswig-Holstein, Lower Saxony, Saxony-Anhalt, Berlin and Brandenburg. However, other areas of Germany are not excluded.

Institutions that are established in training professional or voluntary representatives will offer the education program, including regional departments and associations responsible for legal representatives and a leading education institute (*Institut für Innovation und Praxistransfer in der Betreuung* (ipb))*,* organized by the Federal Association of Legal Representatives (*Bundesverband der Berufsbetreuer/innen* (BdB) *e.V.).* The ipb offers training throughout Germany and is intended to support the evaluation.

The education program will be conducted in the University of Hamburg as well as in the cooperating institutions.

### Eligibility and recruitment

The target group for the intervention is legal representatives, both professional and volunteer. Additionally, data will be assessed from all people with dementia who are represented by one of the participating legal representatives at least for 2 weeks within the 6-month follow-up (hereby referred to as *persons concerned*).

Legal representatives who represent at least one person with dementia (assessed by the legal representative and / or medical diagnosis) are eligible for inclusion. Exclusion criteria are former participation in the PRODECIDE education program (either the whole program or a single module).

Recruitment will be performed consecutively in cooperation with institutions that offer training for professional or voluntary representatives. Institution cooperation includes recruitment and conducting the education program. Appointments for the education program will be made, and invitations will be published, using the e-mail lists, websites, calendar of events, flyers, etc., of the cooperating institutions. The invitation covers information about both the education program (e.g., target group, content, place and time) and the study (e.g., randomized allocation, offers for the control group).

Potential participants will register at the study center (by phone, e-mail or fax). After screening for eligibility (by phone), the legal representatives will receive information leaflets, the consent form and forms for baseline assessment (by mail or e-mail).

### Randomization and blinding

**Fig. 1:**
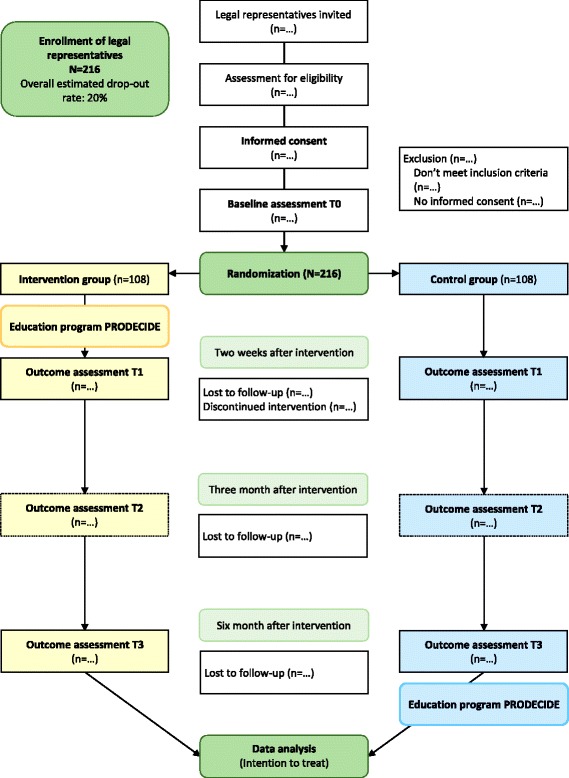
Study Flow

After retrieval of written informed consent and baseline data, participants will be allocated to the intervention group or to the control group, stratified by professionals and volunteers (Fig. 1). To ensure a close balance of entities in each group, randomization will be performed by randomly selected block sizes of four and six. The randomization list will be computer generated by a scientist who is not involved in either the intervention or data collection. An independent person will also perform the allocation. As safeguards, no other person will have access to the list, and the list will be sent by e-mail only in an encrypted format.

Due to the nature of the intervention, blinding of the participating legal representatives and the researchers conducting the education program is not possible. Independent persons and an external biometrician, blinded to the group allocation, will perform data entry and data analysis, respectively.

### Interventions

#### Intervention group (IG)

The intervention comprises a 10-hour education program of four modules that generally is given over 2 days. Module A addresses the decision-making process and introduces the assessment of harms and benefits. The aim is to enhance critical analysis of medical interventions and competencies in informed decision making. The modules B, C and D transmit evidence-based knowledge to the example decisions. Case studies are used to demonstrate practical relevance and to enable participants to take a stand in discussions. The participants receive written education material and evidence-based information leaflets. Two persons (TR, JL) will conduct each training session. Courses will be offered free of charge for the equipment and staff. Participants will have to pay a reduced fee for room rental and catering if necessary. Indeed, charging a low fee might be more effective at increasing the willingness to participate after registration than offering a free-of-charge course.

Both the curriculum and the education material have already been tested for feasibility in the pilot study [34].

#### Control group (CG)

As no equivalent intervention is available, the control group (CG) will receive standard care. After data collection is completed (e.g., after the six-month follow-up), CG participants will be invited to take part in the education program.

### Outcomes

The primary outcome measure is *knowledge*, which is operationalized as an understanding of decision-making processes in healthcare affairs and in setting realistic expectations regarding probabilities of benefits and harms of PEG, PR and AP to people with dementia.

Secondary outcome measures are: 1) legal representatives have obtained sufficient knowledge (using a cut-off of 70% correct answers in the knowledge test); 2) legal representatives have obtained sustainable knowledge, which is measured 6 months after intervention; 3) percentage of persons concerned affected by PEG, FEM or AP during follow up of 6 months; and 4) result of the first decision after intervention regarding PEG, PR and AP for the persons concerned including time to first decision.

### Data collection

In the T0 baseline, data of legal representatives will be assessed according to age, gender, education status, activity status (professional or voluntary representative), time since appointment as legal representative and number of persons concerned. Additionally, baseline data and baseline outcome measurements of persons concerned will be assessed. For this, legal representatives will receive a pseudonymization list, documentation sheets and a standardized written introduction. A pseudonymization number will be assigned to each person concerned with dementia. All data will be given to the study site under this pseudonym. The legal representative, using data extracted from routine documentation, will fill in the documentation sheets. The age range, gender, diagnosis additional to dementia, presence of PEG, number and types of PR, name and dosage of antipsychotics will be assessed by multiple choice and free-response questions. Copies of the routine documentation (e.g., a list of drug prescriptions) will be passed on only if pseudonymization is safeguarded.

At T1, the primary outcome *knowledge* will be assessed (Table 1). To assess understanding and realistic expectations, a novel questionnaire was developed based on Bloom’s taxonomy [40], the *User Manual–Realistic Expectations* [41], the contents of the education program, and the evidence-based information for PEG, PR and AP in people with dementia. The questionnaire comprises 13 items: two items on the understanding of decision-making processes in healthcare affairs; two items on quality and validity of study results; and nine items on realistic expectations regarding probabilities of benefits and harms of PEG, PR and AP to people with dementia (with three items per intervention). All items are multiple choice questions with four choices, with only one correct answer. Questions with more than one answer and unanswered questions will count as a wrong answer. No summarizing score is given if four or more of the 13 questions remain unanswered.

**Table 1:** Data collection

Outcomes	Measures	Follow-up
Knowledge	Questionnaire developed on the basis of evidence-based information and contents of the education program	T1, T3
First decision PEG	Standardised telephone interview	T2, T3
First decision PR	Standardised telephone interview	T2, T3
First decision AP	Standardised telephone interview	T2, T3
Physical restraints	Documentation sheet; medical and nursing records	T3
Antipsychotics	Documentation sheet; medical and nursing records	T3
PEG	Documentation sheet; medical and nursing records	T3

T1 = at the end of / up to 2 weeks after the intervention; T2 = 3 month follow-up; T3 = 6 month follow-up

Legal representatives in the IG will receive the test at the end of the education program. They may complete the test immediately or return it by mail in the following 2 weeks. Participants in the CG will receive the test by mail at the same time.

At T2 (the 3-month follow-up), the results of the first decisions regarding PEG, PR and AP for the persons concerned will be assessed (Table 1). Decisions may have been initiated by legal representatives themselves (e.g., consultation for medication review), by relatives (e.g., worries about agitation) or by health professionals (e.g., required consent for PEG or PR). We define a decision as every consideration about PEG, PR or AP, regardless of the result.

Participants receive a documentation sheet for each intervention at the beginning of follow-up to assess the starting point of the decision-making process, presence of the intervention before and after the decision, reason or trigger for decision-making and changes regarding the intervention. The sheets may be used for personal documentation only or may be sent back to the study center.

Participants will be contacted by phone to ask if they had made a decision regarding PEG, PR and/or AP. If they had made a decision, they will either be reminded to fill out and return the documentation or directly interviewed by telephone to fill out the sheet. The first decision of each intervention will be recorded and classified in one of the four following categories, whereby 0 = no presence and 1 = presence of PEG, FEM and AP:

Category I: 0 ➔ 0 (No PEG / FEM / AP ➔ decision ➔ no PEG / FEM / AP).

Category II: 0 ➔ 1 (No PEG / FEM / AP ➔ decision ➔ new PEG / FEM / AP).

Category III: 1 ➔ 1 (PEG / FEM / AP ➔ decision ➔ still PEG / FEM / AP).

Category IV: 1 ➔ 0 (PEG / FEM / AP ➔ decision ➔ no longer PEG / FEM / AP).

The date of the decision will be documented.

At T3 (the 6-month follow-up), the sustainable knowledge of legal representatives will be assessed using the same test as at T1.

Additionally, the number of PEG, PR and AP interventions in the persons concerned will be assessed (Table 1). The legal representatives will perform the assessment as at T0, filling out the documentation sheets and forwarding any copies of routine documentation. New diagnoses since baseline, the presence of PEG, the number and types of PR, medication data and decisions made about one of these interventions will be documented.

All persons concerned who are represented by one of the participating legal representatives at least for 2 weeks within the 6-month follow-up are eligible. If a person concerned who was assessed at T0 is no longer represented by the participant prior to end of follow-up, a documentation sheet will be filled out. The time of and reason for dropout, as well as the presence of PEG, number and types of PR and medication data at the time of dropout, will be documented. New persons concerned during the study period will be assessed at the time of inclusion and at T3. At the time of inclusion, a documentation sheet will be filled out, including time of inclusion, age range, gender, any diagnoses additional to dementia, the presence of PEG, number and types of PR and medication data.

Participants will receive the knowledge test and documentation sheets by mail or, if requested, by email. Participants in the CG have to submit the documents before subsequently beginning the education program (they may hand them over in person on the first training day).

Participants who had not made all three decisions regarding PEG, PR or AP for a person concerned at T2 will be additionally contacted by phone and asked again if they had made a decision since T2. Results of the first decision of each intervention will be recorded as in T2.

To reach a high compliance rate until T3, participants will receive stamped, addressed envelopes for returning outcome assessments by mail. Submission of all documents to the study center will be also possible by email or fax. If required, participants will be reminded by phone, and any of their questions about data assessment will be answered.

### Data analysis

Data will be entered into a SPSS database and double-checked by student assistants blinded to the group allocation. Simple plausibility checks will be done in the study center before the final statistical analysis by the statistician. Analysis of all quantitative data will use the intention-to-treat principle. Missing values in primary and secondary outcomes will be imputed using simple methods. A dropout analysis comparing baseline parameters between the study population and early dropouts (before T2) will be performed.

All statistical tests are two-sided using a significance level of 5%. Baseline parameters are described by frequency tables, means, standard deviations or quartiles.

The primary outcome is the percentage of correct answers for the knowledge test at T1. Assuming approximate normal distribution, the expected values from the IG and CG will be compared with adjustment for stratified randomization by professionals and volunteers using bifactorial analysis of variance (linear model). Distribution assumptions will be investigated by graphical methods. Furthermore, interactions between intervention and professional status will be included in a secondary model. Different effects depending on the professional status will be discussed.

The secondary outcome of sufficient knowledge (binary) at T1 will be compared between IG and CG using bivariate logistic regression, including IG/CG and professional status as independent variables. Additionally interactions between intervention and professional status will be investigated in a second model.

The sustainability of knowledge will be investigated at T3. The time course of the knowledge between T1 and T3 will be analyzed by fitting a linear mixed model: the dependent variable is knowledge (% of correct answers), and the independent variables are IG/CG, time (T1, T3), interaction between intervention and time, professional status and interaction between intervention and professional status. To adjust for repeated measurements of the legal representatives, random effects are included (covariance pattern model with covariance structure compound symmetry).

Further secondary outcomes are the numbers of persons concerned and the percentage of these persons affected by PEG, FEM or AP (separately) per legal representative at T3. Mean values ± standard deviations will be described for IG and CG at T3 as well as at T0. Linear models will be fitted including IG/CG and initial values at T0 as independent variables.

Finally, the results of the first decision about PEG, FEM or AP by the legal representative after T0 (e.g., after training for IG, or after randomization for CG) will be assessed. Initially, the duration from T0 to the first decision will be described for IG and CG by Kaplan-Meier curves. In case of no decision, durations are censored at the end of observation after 6 months or at dropout. Kaplan-Meier curves between both groups will be compared by the log rank test, as long as there are no clear deviations from the proportional hazard assumption in the graphics. Four categories of decisions will be described by frequency tables.

### Sample size

No information was available for the primary outcome from previous studies. The assessment instrument was pretested with a before-after design to roughly estimate the expected intervention effect in the primary outcome of knowledge (given as a percentage of correct answers). The knowledge test was revised after three courses with a total of 18 legal representatives. Afterwards, two pilot courses with 16 participants were used to estimate the effect size of the intervention. Fifteen datasets before intervention, and 12 after intervention, were included in the analysis.

A common standard deviation of σ = 0.17 can be assumed in IG and CG. A mean difference of 0.085 between IG and CG can be detected by a power of 90% by the two-sided t-test, using a significance level of 5% based on a sample size of 86 per group (172 overall). Including a maximum dropout rate of 20%, an overall sample size of 216 is planned. The pilot study estimated a larger effect (0.38). Because of the low sample size, and considering a possible bias in the pilot study, the planned sample size is higher than theoretically necessary. It corresponds to a medium effect of 0.5*σ, as suggested by Cohen.

With six to ten participants per training session, the education program will be offered approximately 24 times (12 times per group).

### Monitoring

A data monitoring committee will not be necessary, as the clinical trial does not involve a high-risk intervention and participants do not belong to a vulnerable population. We do not expect adverse events or other unintended effects of the intervention. During the entire study period, participants will have the possibility to contact the training experts.

### Process evaluation

To allow the study results to be generalized and to support future implementation, a comprehensive analysis of the underlying processes of this complex intervention is indispensable [42]. Barriers and facilitators of implementation should be assessed. Additionally, the high quality of the education program should be ensured. We will focus on parameters such as recruitment, reasons for participation or non-participation, intervention fidelity, structure- and process-related factors, attitudes toward the intervention, response of individuals and organizations and unintended consequences [42, 43].

Mixed methods will be applied [44] according to the MRC guidance for process evaluation of complex interventions [42].

Structured documentation will be used to assess data of recruitment and intervention fidelity (e.g. recruitment process; numbers of persons invited, responses and participants in each module; location, time and duration of the training; completeness of modules and reasons for deviations; and unexpected difficulties).

Recruitment and conducting the education program will be performed in cooperation with the participating institutions, which are experienced in offering training. Standardized interviews will be conducted with coordinators from these institutions to explore barriers and facilitators of implementation.

Feasibility and acceptance of the education program will be assessed at the end of each training session in a feedback round. All participants will be invited to take part, and statements from the feedback round will be documented.

In the IG (theoretical sampling), semi-structured interviews will be conducted. Relevant factors for acceptance and usability of the educational contents and materials may be further assessed. In particular, the use of educational contents in daily routines will be explored. Participants will be asked to describe a decision-making process, the roles of persons involved and their perceived changes in this process after the education program. If participant consent is provided, interviews will be audio recorded and then transcribed.

To gain further inside into behavioral changes, intermediate outcomes (e.g. number and content of conversations with healthcare professionals) will be assessed in a random sample of legal representatives (from both IG and CG).

Quality standards will be derived from existing standards in the field of continuing education [45, 46]. For PRODECIDE implementation, the development of objective, measurable criteria is a key aim (e.g. “Percentage of participants who completed all modules.”). Quality standards will be predefined and then refined and completed during process evaluation.

Data will be collected at various time points. An iterative process of collecting and analyzing qualitative data will allow exploring unexpected aspects in further interviews [42]. Data will be analyzed in accordance with the method of collection [44]. Descriptive statistics will be used for quantitative data. For qualitative data, a qualitative content analysis according to Mayring will be performed [47].

### E-learning concept

A concept for e-learning modules will be developed and pilot-tested. E-learning supports self-regulated learning and allows individuals to access the education program without time or location restrictions. This thereby allows the educational offering to fit the personal and professional requirements of the users.

### Development of e-learning modules

To develop the e-learning modules, the content of the education program will be transferred into a learning management system such as OLAT (*Online Learning And Training*), an open-source learning management system. As OLAT supports a variety of online courses and web applications, it is suitable for implementing a web-based education program [48].

OLAT comprises specific tasks to realize complex learning and teaching scenarios. Tools can be used to create and edit content (Wiki), to communicate (e-mail, forums) and to manage course contents in different formats. Different medial preferences can be addressed.

Synchronous or asynchronous interaction with and between participants is possible (chats and virtual classrooms or forums, respectively). Additionally, tools to assess the individual learning progress can be integrated. Early in the development process, quality criteria will be taken into account for the use of online courses and web applications, such as consistency, user control, ease of learning, flexibility, error management, user help and user guidance responsiveness [49]. A test module will be developed to assess usability.

### Usability test

In a qualitative approach, the usability of the educational content presentation in online modules will be tested. Content comprehensibility was tested previously [34] and is of secondary importance.

Recruitment will be consecutive, and the number of participants will be determined by theoretical sampling, based on age, gender, activity status and the participant’s knowledge and experiences relevant to the investigation [47]. Therefore, potential participants will be asked to self-assess their IT skills. Both professional and voluntary representatives will be included. A sample size of 15 participants is anticipated to reveal 90–97% of usability problems [50]. Iterative recruitment will be performed until data saturation is achieved.

To explore and understand usability problems, a concurrent think aloud method will be applied [51, 52]. Participants will be observed while they interact with the test module and will be asked to think aloud while they work. All aspects of usability (e.g., navigation, design and layout) will be addressed. Additionally, semi-structured interviews will be performed to better understand any problems encountered and to ask for suggestions for improvement.

Records of observations and interviews will be summarized. Qualitative content analysis according to Mayring will be performed [47]. Data will be paraphrased and, based on usability criteria [49], will be categorized and interpreted. Revision and further testing is possible as long as serious usability problems are identified.

### Ethics

Ethical approval for the proposed project was received by the ethics committee of the German Society of Nursing Science (*Deutsche Gesellschaft für Pflegewissenschaft*) on 1 October 2015. The ethics committee will be informed of relevant modifications or additions to the course of study.

The following ethical aspects will be considered: the included legal representatives are capable of making decisions and are free to participate; the interested legal representatives will receive detailed written information before participation; written informed consent will be received; the corresponding documents have previously been revised by the ethic committee; data from persons concerned will be collected pseudonymized and indirectly via the legal guardians; no negative effects for the participants or for persons concerned are expected; data protection will be taken into account, to the greatest extent possible; and temporary data storage of personal information will be done in an encoding list.

The *Ethical Principles for Medical Research Involving Human Subjects* (WMA Declaration of Helsinki [53]), the guidance for *Good medical practice* (General Medical Council [54]) and the recommendations for safeguarding *Good Scientific Practice* (*Deutsche Forschungsgemeinschaft* [55]) will be followed in the proposed project.

### Dissemination

All results of the study (including negative ones) will be published in international and open-access journals and presented at meetings and congresses. All participants will receive an abbreviated version of the final report written for laypersons.

After study completion, adjusted data will be stored and made publicly accessible via a specialized database. To meet the DFG requirements for data sharing [56], data will be published and maintained in the so-called “datorium,” a service of the GESIS – Leibniz- Institute for the Social Sciences [57]. This will also fulfill the requirements for sharing clinical trial data of the US *Institute of Medicine* [58, 59].

We believe that a long-term dissemination and implementation of the PRODECIDE education program for training and advanced training offers for legal representatives is essential. Legal representatives frequently have to make decisions together with, or for, a highly vulnerable population group. The PRODECIDE education program is exemplary of an evidence-based, modularly structured vocational training or university curriculum. Module A constitutes a comprehensive approach and a methodological basis that can be used for healthcare-related decisions in other somatic and psychiatric diseases.

Conditions for a sustainable implementation of the proposed program have been met. The ipb, which is part of the BdB (a professional association for legal representatives in Germany with more than 6500 members) and which offers training throughout Germany, has expressed interest in making the PRODECIDE program a standard offer in its training. Additionally, associations in Hamburg and other federal states of Germany that provide support for voluntary representatives have indicated interest in offering our program. Sustainable implementation will be further ensured by the parallel development of an e-learning concept.

## Discussion

In this RCT, we will evaluate the efficacy of the PRODECIDE education program in the context of training for legal representatives.

The PRODECIDE study has several strengths. Legal representatives will be allocated to the intervention or to the control group using a computer-generated randomization scheme. An independent person not involved in either the study or data collection will perform the allocation. Data entry will be performed blinded. Various approaches will be taken to reduce missing data and dropouts. These strategies were adapted from previous RCTs with very low attrition rates [20, 60, 61]. For analysis, the intention to treat principle will be used.

The study limitations include the fact that, due to the nature of the intervention, neither participants nor researchers conducting the education program and data collection will be blinded. Likewise, it is not possible to safeguard either the inclusion of all eligible persons with dementia or the correct documentation of PEG, PR and AP for all persons concerned. Only the legal representatives will perform the assessment for eligibility, and they will give the data of the persons concerned to the study center under a pseudonym. We assume that the error ratio will be comparable in IG and CG. Nevertheless, it is possible that enhanced knowledge in the IG leads to a more correct documentation and to a bias of results. Data collection will be verified by using medical and nursing records if possible. Finally, legal representatives are well connected, for instance through office partnerships or supervision sessions, such that “contamination” from the intervention to the control group by sharing educational materials is possible. However, as the training is both hands-on and in-depth, we do not expect that the CG has an improved knowledge solely on the basis of written educational materials.

At the end of this study, information about the efficacy of the PRODECIDE education program, the usability of e-learning modules and processes that interfere with or promote a successful implementation into the regular training curricula for legal representatives will be available.

## Abbreviations

AP: antipsychotic drugs
BdB: Bundesverband der Berufsbetreuer/innen
CG: control group
DFG: Deutsche Forschungsgemeinschaft
IG: Intervention group
ipb: Institut für Innovation und Praxistransfer in der Betreuung
OLAT: Online Learning And Training
PEG: percutaneous endoscopic gastrostomy
PR: physical restraints
RCT: randomized controlled trial
